# Donor funding for family planning: levels and trends between 2003 and 2013

**DOI:** 10.1093/heapol/czy006

**Published:** 2018-03-09

**Authors:** Christopher Grollman, Francesca L Cavallaro, Diane Duclos, Victoria Bakare, Melisa Martínez Álvarez, Josephine Borghi

**Affiliations:** 1 Department of Global Health and Development, London School of Hygiene and Tropical Medicine, 15–17 Tavistock Place, London WC1H 9SH, UK,; 2 Department of Infectious Disease Epidemiology, London School of Hygiene and Tropical Medicine, London, UK and; 3 School of Medicine, King’s College London, Guy’s Campus, Great Maze Pond, London SE1 9RT, UK

**Keywords:** Family planning, health financing, donor policies, agenda setting

## Abstract

The International Conference on Population and Development in 1994 set targets for donor funding to support family planning programmes, and recent initiatives such as FP2020 have renewed focus on the need for adequate funding to rights-based family planning. Disbursements supporting family planning disaggregated by donor, recipient country and year are not available for recent years. We estimate international donor funding for family planning in 2003–13, the period covering the introduction of reproductive health targets to the Millennium Development Goals and up to the beginning of FP2020, and compare funding to unmet need for family planning in recipient countries. We used the dataset of donor disbursements to support reproductive, maternal, newborn and child health developed by the Countdown to 2015 based on the Organization for Economic Cooperation and Development Creditor Reporting System. We assessed levels and trends in disbursements supporting family planning in the period 2003–13 and compared this to unmet need for family planning. Between 2003 and 2013, disbursements supporting family planning rose from under $400 m prior to 2008 to $886 m in 2013. More than two thirds of disbursements came from the USA. There was substantial year-on-year variation in disbursement value to some recipient countries. Disbursements have become more concentrated among recipient countries with higher national levels of unmet need for family planning. Annual disbursements of donor funding supporting family planning are far short of projected and estimated levels necessary to address unmet need for family planning. The reimposition of the US Global Gag Rule will precipitate an even greater shortfall if other donors and recipient countries do not find substantial alternative sources of funding.

Key Messages

- Donor funding supporting family planning programmes is far lower than needed to meet past targets or current needs.
- The majority of past funding has come from the United States, suggesting the reintroduction of the Mexico City policy may leave a huge shortfall even in current levels of funding. Following the previous reimplementation of the Mexico City policy in 2001, other donors did not increase disbursements to offset lost US funds.
- Funding for family planning is increasingly targeted toward recipient countries with higher national levels of unmet need for family planning.

## Introduction

The ability to control the number and spacing of one’s children is a key reproductive right, alongside the right to safe and effective care in pregnancy and childbearing (Cook and Fathalla 1996). By reducing the number of births and unsafe abortions, family planning also reduces maternal mortality and morbidity (Ahmed *et al.* 2012). Family planning is at the centre of a number of global initiatives. The 1994 International Conference on Population and Development Programme of Action (ICPD PoA) described the major components of ‘population and reproductive health programmes’ as family planning, basic reproductive health services, sexually transmitted infections/HIV, and research and policy. Although the Millennium Development Goals (MDGs) originally did not refer to reproductive health, MDG target 5B to ‘achieve universal access to reproductive health’ was advocated for (UN Millennium Project 2005) and added in 2005.

Since the beginning of large-scale family planning programmes in the 1960s, bilateral donors—particularly the USA—and multilateral agencies have provided much of the funding for programmes in low- and middle-income countries (Population Reports 1983; Sinding 2007). This is still the case—the United Nations Population Fund (UNFPA) and Netherlands Interdisciplinary Demographic Institute (NIDI) (UNFPA and NIDI 2014) report that ‘most developing countries’ continue to be ‘dependent on the international donor community to finance population activities’ (2014, p. 36).

There were concerns over reduced and volatile donor (and domestic) funding in the 2000s (Cleland *et al.* 2006; Ross *et al.* 2007; Petroni 2009), and calls for increased investment to meet demand for contraception (Alkema *et al.* 2013). The largest recent initiative, the FP2020 conference in 2012, aimed to refocus policy-makers’ attention on family planning.

The estimated cost of supporting family planning in low- and middle-income countries is substantial. In its cost estimations for ‘developing countries’ and ‘countries with economies in transition’, the ICPD PoA assigned over 60% of projected costs to the family planning component, amounting to targets of $5.6 b of donor funding for family planning in 2005 and $6.2 b in 2010 (in 2013 USD) (UNFPA 2004). More recently, the Guttmacher Institute estimated the cost of meeting 100% of need for family planning in ‘developing countries’, from all funding sources, at $7.2 b in 2008 and $9.3 b in 2014 (in 2013 USD) (Singh *et al.* 2014).

In recent years a number of initiatives have sought to track global funding flows to support progress towards reproductive, maternal, newborn and child health, including family planning (Hsu *et al.* 2013; UNFPA and NIDI 2014;Fan *et al.* 2017; Institute for Health Metrics and Evaluation 2017). Hsu *et al.* (2013) estimated disbursements for family planning at $289 m in 2009 and $299 m as part of the Countdown 2015 initiative in 2010 (2010 USD). UNFPA and NIDI estimates suggest that <$1 b was disbursed for family planning from bilateral donors and through the UN system in each year up to 2010 (2013 USD, our conversion) (UNFPA and NIDI 2014). A recent study by Fan and colleagues using Organisation of Economic Co-operation and Development’s (OECD) CRS records reported under the family planning purpose code found that over the period 2004–14, donors disbursed $5.9 b for family planning (2013 USD) (Fan *et al.* 2017). The estimates from UNFPA/NIDI, Fan and colleagues and IHME do not provide a detailed breakdown of family planning funding by donor, recipient country and year. Previous estimates from the Countdown initiative (Hsu *et al.* 2013; Arregoces *et al.* 2015) did not cover the whole period 2003–13 and were made prior to the completion of that project and the updating of its dataset, which is now publicly available (Grollman *et al.* 2017a,b).

Donors consider many factors in making decisions about where to target their funds, among which need is only one (van Dalen and Reuser 2006), and one for which there are many possible indicators (Dixon-Mueller and Germain 2007). The ICPD PoA emphasizes unmet need for family planning—percentage of women at risk of pregnancy who do not want to become pregnant and who are not using contraception—as a preferred indicator (UNFPA 2004).

Fan *et al.* (2017) also investigated whether in 2012–14, donors prioritized family planning disbursements to recipient countries according to several indicators of national-level need (contraceptive use, population growth, maternal mortality, gender inequality, unmet need and demand satisfied). They found that recipient countries with greater need, across all indicators, were often under-prioritised. They also found a moderate association between funding and unmet need for family planning (*R*^2^ = 0.35). Leading family planning donors emphasize unmet need in strategy and research documents (UK DFiD 2011; USAID 2012; Kingdom of the Netherlands 2014), suggesting it is reasonable to expect this indicator to play a role in decisions about targeting funding.

This article has two objectives: (1) to provide annual estimates, by donor and recipient country, on the levels and year on year trends of funding for family planning from 2003 to the beginning of FP2020-related disbursements in 2013; (2) to assess whether funding is targeted to countries with highest unmet need for family planning and whether this has changed over time.

## Methods

### Data sources

We used the Countdown to 2015 aid-tracking dataset (Grollman *et al.* 2017a) to provide estimates of donor funding for family planning from 2003 to 2013. This dataset contains disbursement records of ‘official development assistance’ and private grants (together called ‘ODA+’) reported by donors to the OECD Creditor Reporting System (CRS) and data directly from Global Vaccine Alliance (GAVI) for 2003–06. Donors reporting are bilateral donors (the OECD countries and several others), multilateral institutions (including development banks and the European Union), global health initiatives (GAVI and the Global Fund) and a private foundation (Bill and Melinda Gates Foundation). We avoided double counting by using the OECD definitions of donor type, which exclude from bilateral donations core contributions to multilateral agencies and ascribe spending by multilaterals to those agencies alone. The Countdown dataset only includes donors reporting to CRS—this omits non-governmental organisations and most foundations, as well as middle-income bilateral donors such as China, India and Brazil. It also excludes domestic spending, although the proportion of family planning funding that comes from domestic resources is often low (UNFPA and NIDI 2014). Donors have been vital for successful family planning programmes (Olson and Piller 2013) and the transition from donor-supported programmes to domestically financed programmes can leave substantial shortfalls (Drake *et al.* 2010).

In the Countdown ODA+ dataset, described in detail elsewhere (Grollman *et al.* 2017b), the CRS disbursement records from all sectors were coded for relevance to reproductive, maternal, newborn and child health (RMNCH) across several activities including family planning. Records were manually reviewed and assigned one of a set of 27 activity codes reflecting their benefit to RMNCH. The family planning code was assigned to records that met the following definition:

Project is oriented to family planning including the provision of and counselling in contraceptive commodities, abortion services, infertility drugs and procedures, and information, education and communication (IEC) activities that support or promote family planning (Grollman *et al.* 2017a).

To assess targeting to unmet need for family planning, we used estimates of unmet need from the United Nations Department of Economic and Social Affairs (UNDESA Population Division 2015). These estimates exclude Micronesia, Mayotte and Seychelles, for which data on unmet need were unavailable and to which total disbursements across the 11-year period were <$1 m (0.02%). We chose unmet need over other family planning-related metrics because it takes account of the reproductive desires of individuals, in contrast to measures such as fertility rates or contraceptive prevalence (Dixon-Mueller and Germain 2007), reflecting the human rights-based focus of contemporary family planning programmes (Hardee *et al.* 2014). It is a metric used and referred to more widely than proportion of demand satisfied, which also takes account of users’ desires. As well as its promotion in the ICPD Programme of Action, unmet need has been widely adopted (Ali *et al.* 2014; Afnan-Holmes *et al.* 2015) and forms part of the indicator in the Sustainable Development Goals on the ‘Proportion of women of reproductive age (aged 15–49 years) who have their need for family planning satisfied with modern methods’ (UNDESA Statistics Division 2016). We obtained population data from the World Bank (2016).

All data sources were publicly available and no ethical clearance was needed to conduct this analysis.

### Data analysis

To estimate annual funding levels for family planning, we summed disbursements in the Countdown dataset for records assigned the family planning activity code. We also calculated total annual disbursements by donor and recipient country overall and per woman of reproductive age, and graphed year on year disbursements by recipient country year- to assess volatility. Disbursements made to regional recipients (e.g. ‘Asia, regional’) or to ‘Bilateral, unspecified’ were assigned to recipient countries proportionally to their year-specific share of direct disbursements within the region (for regional disbursements), or to all recipients (for ‘Bilateral, unspecified’) (Grollman *et al.* 2017a). We examined family planning funding as a share of funding to reproductive, maternal, newborn and child health presented previously (cite Grollman *et al.*2017a). All values are in constant 2013 US dollars.

To assess whether funds for family planning were targeted to recipient countries with the highest unmet need for family planning, we compared the distribution of funding disbursements to that of country-level unmet need using concentration curves. We plotted the cumulative share of unmet need by recipient country ordered from highest to lowest national level of unmet need, against the cumulative share of family planning disbursements received. Sections of the curve steeper than the line of equality represent countries that receive a disproportionately large share of funding compared to their share of unmet need; sections flatter than the line of equality represent countries receiving a disproportionately small share. A curve above the line of equality indicates overall targeting to higher national levels of unmet need. We calculated concentration curves in 2003, 2008 and 2013 to examine whether targeting to unmet need changed over time.

## Results

### Levels and trends for family planning disbursements

**Figure 1.**
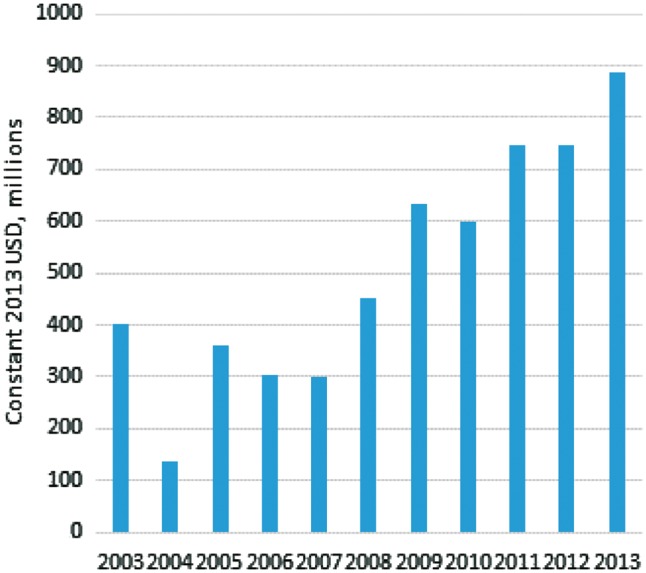
Total ODA+ disbursements for family planning

Across 2003–13 a total of $5566 m was disbursed in 9913 transactions (Figure 1), comprising $3452 m to named recipient countries (62%), $1906 m to unspecified recipient countries (34%) and $209 m to recipient regions (4%).

**Figure 2.**
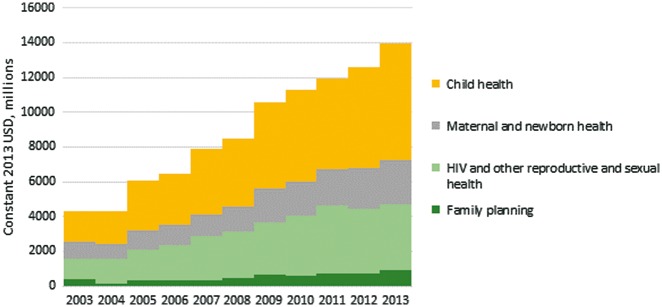
ODA+ disbursements for family planning, HIV, other reproductive and sexual health activities, maternal and newborn health, and child health

Disbursements to family planning fell between 2003 and 2004, from $402 m to $137 m, before rising to $360 m in 2005 and staying around $300 m for 2006–07. Disbursements increased from $452 m in 2008 to a high of $886 m in 2013. There was similar growth for overall disbursements supporting RMNCH, and the proportion supporting family planning was fairly stable: 4–6% of total disbursements for RMNCH, except in 2003 (9%) and 2004 (3%) (Figure 2). Throughout the period, around 30% of RMNCH disbursements were for HIV and other reproductive and sexual health, 18% were for maternal and newborn health and 46% were for child health.

**Figure 3.**
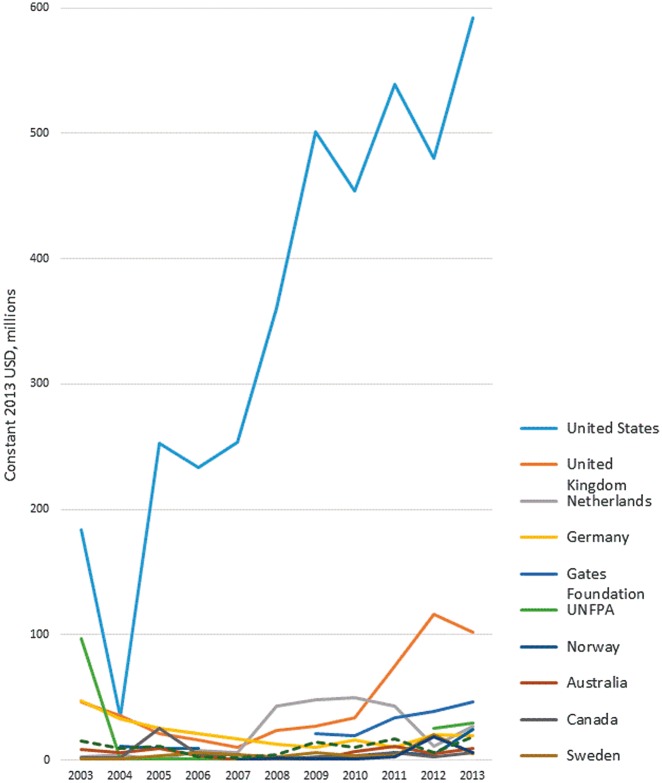
ODA+ disbursements for family planning to all recipient countries, by donor

Almost all disbursements (93%) came from bilateral donors. The largest donor by far throughout the study period was the USA, which provided 70% of total funds to family planning across the period 2003–13. The level of funding from the United States reduced dramatically from $183 m in 2003 to $34 m in 2004, before rising to $253 m in 2005. In 2003, the UNFPA was the second-largest donor. However, their disbursements dropped to almost zero in 2004 until 2012. The next largest donors were the United Kingdom (9.1% of total disbursements), the Netherlands (4.3%) and Germany (4.2%) (Figure 3, Supplementary Table S2).

**Figure 4.**
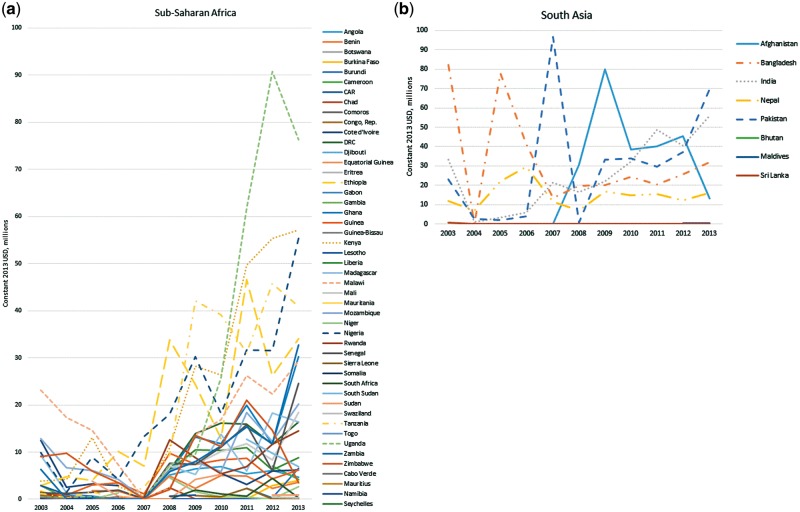
(a) ODA+ disbursements for family planning to recipient countries, Sub-Saharan Africa. (b) ODA+ disbursements for family planning to recipient countries, South Asia. (c) ODA+ disbursements for family planning to recipient countries, All other recipient countries

The recipients of the 10 largest disbursements in aggregate over the 11 years were Bangladesh, Philippines, Pakistan, India, Uganda, Kenya, Haiti, Afghanistan, Egypt and Nigeria. The 10 smallest total disbursements were to Mauritius, Montenegro, Belarus, Oman, Bhutan, Seychelles, Republic of Congo, Micronesia, Panama and Equatorial Guinea. India and the Philippines were among the largest recipients for all or most years, while others saw dramatic year-on-year fluctuations. For example, disbursements to Pakistan were <$5 m in 2006 and 2008, and almost $100 m in 2007. Disbursements to Malawi fell from over $20 m in 2003 to almost zero in 2007, before returning to over $20 m in 2011 (Figure 4).

Disbursements per woman of reproductive age have increased slowly over time: in 2003, 19 countries received at least $2 per woman of which 6 received more than $6 per woman. In 2013, these numbers were 25 and 8, respectively. However, this growth was limited and uneven: 24 of 53 countries with >20% unmet need continued to receive <$1 per woman of reproductive age in 2013. In each year, 68–94% of recipient countries received <$1 per woman of reproductive age, regardless of their level of unmet need; 43% of countries received <$1 per woman of reproductive age in every year (Supplementary Table S3).

### Relationship with unmet need for family planning

**Figure 5.**
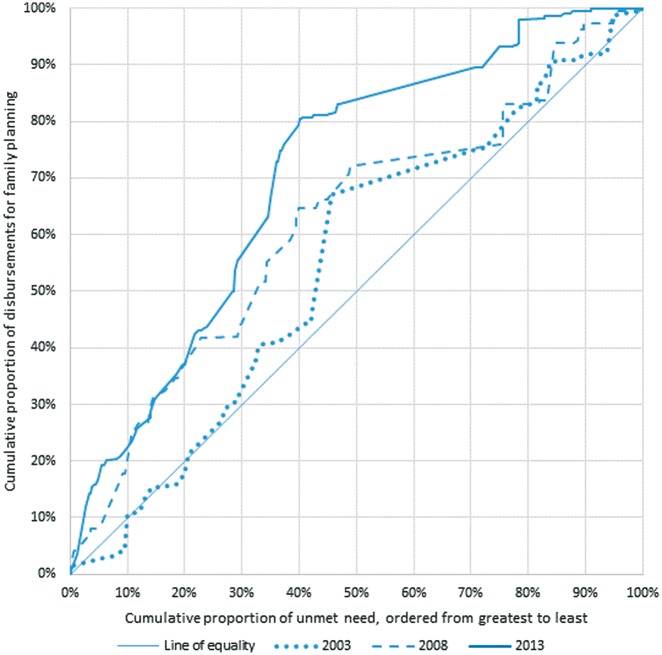
Targeting of ODA+ disbursements for family planning to unmet need

Countries with higher unmet need received disproportionately more funding for family planning across the period than those with lower unmet need, as demonstrated by the concentration curve lying above the line of equality in all three years. This concentration has increased over time: between 2003 and 2013, family planning disbursements became more concentrated among countries with higher levels of unmet need (Figure 5, Supplementary Table S3). For example, in 2003 the 20% of unmet need in the countries with highest national levels of unmet need received around 18% of total disbursements; this proportion increased to 36% in 2008, and 37% in 2013. The proportion received by the countries with the lowest national levels of unmet need fell from 17% in 2003 and 2008 to 2% in 2013.

## Discussion

Over $5.6 b of ODA+ was disbursed to support family planning between 2003 and 2013. Despite initial declines in disbursement levels between 2003 and 2007, there was a substantial increase between 2008 and 2013. The 18% increase in funding between 2012 and 2013 may partly be an initial effect of the FP2020 initiative following the 2012 London Summit. FP2020 reported a 21% increase in bilateral disbursements from 2012 to 2013, similar to our 18%, and reported a further 9% increase from 2013 to 2014 (FP2020 2015).

The levels of funding we report for 2009 and 2010 are roughly double the levels previously reported using Countdown data (Hsu *et al.* 2013). This is partly due to additional funds for those years being reported late by donors, and may also be affected by retrospective changes made to previously coded data by the Countdown initiative (Grollman *et al.* 2017b), ensuring more complete coverage of family planning disbursements in the dataset used in our analysis. The overall trend between 2003 and 2012 in our data is consistent with the trend for donor funding to family planning in the Resource Flows reports published by UNFPA/NIDI, although UNFPA/NIDI found a greater increase, from $498 m in 2003 to $1173 m in 2012 (in 2013 USD) compared with $402–$748 m in our data. The levels cannot be directly compared due to methodological differences between the exercises—particularly that the Resource Flows project includes data from more sources (UNFPA and NIDI 2014). Our results also follow the same trend for family planning funding as reported in the 2016 report on Development Assistance for Health by the Institute for Health Metrics and Evaluation (IHME), although the IHME figures are higher for every year, being over $1bn (2015 USD) in 2009 and 2011–13 (Institute for Health Metrics and Evaluation 2017). IHME relies on a key word search to identify family planning projects, and includes other sources of data, capturing contributions of more donors—1000 foundations and over 600 NGOs, which are not included by Countdown. These additional contributions appear to more than offset the effect of excluding funds to unspecified recipients (which Countdown allocates to named recipient countries proportionally to their share of direct funds) and excluding general budget support (a proportion of which is included by Countdown). The biggest difference between the two datasets may be in the approach to currency conversion and inflation, where the respective approaches can lead to vastly different estimates, as outlined in a forthcoming detailed comparison of the Countdown and IHME tracking methods (Pitt *et al.* 2017).

Our estimates of overall funding and largest donors and recipients is also very similar to the estimates recently published by Fan and colleagues for 2004–14 (Fan *et al.* 2017), although Fan and colleagues focussed on the relationship between funding and need and did not give a full breakdown of annual funding by donor and recipient country.

Although estimates of the level of funding required to meet family planning need vary, even the $886 m disbursed in 2013 is several billion dollars short of the projections of annual resource needs from the ICPD Programme of Action ($6 b) and UNFPA/NIDI ($5 b) (Singh *et al.* 2014).

ODA+ to family planning relies primarily on a small group of bilateral donors, led by US funding, and with almost zero funding from multilateral sources (Figure 3). The stagnant funding from the USA between 2003 and 2007, and particularly the drop in funding in 2004, are consistent with the re-introduction in 2001 of the ‘Mexico City policy’ or ‘Global Gag Rule’, banning US funds to groups that provide information or services relating to abortion (Crane and Dusenberry 2004). Disbursements from the next largest donors also stagnated and declined in this period.

Our analysis suggests substantial volatility year-to-year in funds to many recipients, which is a recognised problem with donor financing (Hamann and Bulir 2001). Volatility can cause problems with planning and budgeting programmes, including meeting staff and commodity costs in the health sector (Lane and Glassman 2007; Juliet *et al.* 2009) and weakening family planning programme effort (Ross *et al.* 2007). However, we are cautious in interpreting this year-to-year variation as necessarily indicating volatility, as large disbursements in a single year could cover several years of service provision and may not mean volatility in funds available to programmes.

We found that donor funding is increasingly concentrated toward countries with higher national levels of unmet need for family planning, with countries with lower levels of unmet need getting a reduced share of funds over time. This would be consistent with the emphasis on unmet need found in documents from the USA, UK and Dutch governments (UK DFiD 2011; USAID 2012; Kingdom of the Netherlands 2014). It is also consistent with Fan *et al.* (2017) finding (presented in their supporting information) that disbursements for family planning in 2012–14 were moderately correlated with number of women with unmet need. Health service programme strength often affects unmet need (Wulifan *et al.* 2016), and low national levels of unmet need may indicate relatively strong national family planning programmes, meaning that targeting donor funding to countries with higher levels of unmet need may be appropriate. However, we do not have any indicator of national programme strength. In the field of health more broadly, many factors affect donors’ decisions, including disease burden, recipient country income and capacity to absorb funds (Gottret and Schieber 2006; Ravishankar *et al.* 2009; Esser and Keating Bench 2011). We are therefore cautious in drawing conclusions about the basis for donors’ decisions about where to give funds. Moreover, sustained donor funding for a strong family planning programme may help some countries maintain lower levels of unmet need, and many areas of spending affect the success of family planning programmes (Daly 1983).

The Countdown ODA+ dataset has a detailed coding scheme that is straightforward to understand and conceptually coherent (Pitt *et al.* 2017), and the CRS, on which it is based, has several advantages, including a consistent reporting framework and the fact that the donors themselves report and agree on all funding reported. Our approach suggests high accuracy in manual review of records to create the Countdown ODA+ dataset, and the inclusion of records from all aid sectors helped identify resources for family planning not identifiable using CRS purpose codes only—disbursements reported by donors solely using the CRS purpose code for family planning (13030) amounted to $4, 832 m, over $700 m less than our estimate (data not shown).

Our analysis has several important limitations. The funding targets set in the ICPD are based on assumed future levels of family planning need and levels of domestic funding, which may not be accurate; however, the ICPD estimates are broadly consistent with more recent estimates of funding need, and the discrepancy between estimated and actual funding levels means that there would be a shortfall even if the ICPD had greatly overestimated future need. Countdown ODA+ data were only available up to 2013, limiting our understanding of more recent trends in disbursements. It is also likely that there were disbursements in the Countdown ODA+ dataset that were described as benefitting maternal or reproductive and sexual health, but would have included direct provision of family planning services (Petroni 2009; Grollman *et al.* 2017a).

Furthermore, a narrow focus on family planning separated from broader sexual and reproductive health also means we do not consider funding for broader activities relating to reproductive justice and sexual and reproductive health and rights that affect effective access to family planning services (Gilliam *et al.* 2009). Together with the omission from the Countdown ODA+ dataset of donors not reporting to the CRS, this means that the present figures should be viewed as a conservative estimate of funding to support family planning. Moreover, donor funding is only one component of the total resources available for family planning in a country, alongside domestic public and private financing.

We examined targeting of disbursements to national levels of unmet need for family planning; although a widely used measure, the extent to which this definition of unmet need captures latent demand for family planning services is unknown. Women who meet the definition of unmet need may not wish to use contraception even if accessible for a number of reasons including low acceptability of contraceptive use or side-effects, which is likely to vary across countries, implying our ranking of national unmet need may not coincide with need that can be met (Sedgh *et al.* 2016). Moreover, it is not known how closely unmet need is a proxy for programme strength, which might be a better basis on which to make targeting decisions.

## Conclusions

Our analysis has shown a substantial increase in family planning disbursements from 2008, reaching nearly $900 m in 2013; nonetheless, these disbursements fall far short of the estimated funding required to fulfil the unmet need for contraception in low- and middle-income countries. Unmet need remains high in many countries, but many still receive <$1 of donor funding for family planning annually per woman of reproductive age. Although our database did not include domestic funding or funding from other private foundations, it is likely that a shortfall of several billion dollars remains. To reduce unmet need and meet the targets in the Sustainable Development Goals and FP2020 requires sustained increases in funding, together with ongoing monitoring and reporting on funds.

The shortfall is likely to increase after the incoming US administration introduced an expanded Global Gag Rule in January 2017 (President of the United States 2017). Low- and middle-income recipient countries that want to maintain access to family planning services or expand to meet unmet need may need to prioritize other sources of funding, including making family planning a greater priority within domestic budgeting. The role of FP2020 in encouraging domestic resource mobilization is positive in this regard. Donors can both help meet their commitments and support autonomous priority setting through increasing general budget support in line with the Paris Declaration on Aid Effectiveness. But the scale of the gap abruptly caused by the reduction of US funds is unlikely to be met through domestic spending alone. Following the US policy announcement, other donors led by the Netherlands established the She Decides initiative, which has generated $300 million in pledged new funding (Government of the Netherlands 2017), but this falls short of US disbursements for any single year in the period 2005–13.

It is possible that demanding that funds for specific activities be easy to identify helps perpetuate practices of vertical programming, undermining efforts to increase partnership and integration across broader fields of development cooperation and healthcare (Storeng and Behague 2016). Nonetheless, some form of high-level tracking of disbursements is valuable in promoting overall donor accountability. This tracking suffers from a lack of agreement on methodology (Pitt *et al.* 2017) and therefore comparability. The approach of the International Aid Transparency Initiative may enhance the timeliness of analyses, although the reliance on CRS purpose codes may be a conceptual drawback. Tracking exercises such as this cannot provide information on the appropriateness of funding for meeting specific goals or needs, which must instead be assessed by ongoing programme evaluation in recipient countries (Lipsky *et al.* 2016). Such work may provide the basis for future research on the relationship between donor funding and the strength of family planning programmes. As determinants of need are complex, an effect of ODA financing would be difficult to isolate and it is unlikely that there would be a single answer across all settings. Such analyses might best be conducted at the national or sub-national levels (Sidze *et al.* 2013). Finally, our findings suggest serious volatility in family planning funding to many recipient countries. Future research should investigate the reasons for large differences in year-on-year disbursements, whether this volatility is reflected in funding available to programme managers and planners and what effect it has on programme sustainability.

## Supplementary data

Supplementary data are available at *Health Policy and Planning* online.

*Conflict of interest statement*. None declared.

## Supplementary Material

Supplementary Table 1

Supplementary Table 2

Supplementary Table 3
