# Supplementary material for: Donor funding for family planning: levels and trends between 2003 and 2013
Source: Health Policy Plan. 2018 Mar 9;33(4):574–82. doi: 10.1093/heapol/czy006 (PMC5894079; doi:10.1093/heapol/czy006)
Supplement: Supplementary Table 1 [file czy006_supplementary_table_1.docx]

Supplementary Table 1: Key terms for investigating aims of funding

Grey = no results. % = wildcard for any number of characters. [eé] = search for term with either character in that position. * = identified through manual review.

| Category | **English** | | **French** | **Spanish** | **German** | | **Dutch** |
| --- | --- | --- | --- | --- | --- | --- | --- |
| **Family planning - generic** | family plann  FP  fmaily planning | family planing  familyplanning | planification familiale  planning familial  planing familial  PF | planificaci%familiar | Familienplanung | | geboorte regeling  geboorte planning |
| **Contraception** | contracept  contrcept  condom  injectable  hormonal  social marketing* | condom  commodit  long acting reversible  pregnant control*  preventing teenage pregnancy* | pr[eé]servatif  contraceptif  contraceptive  r[eé]versible  longue dur[eé]e | anticonceptivo  anticoncepción  contracepción  anticonceptivo  contraceptivo | Verhütung  Empfängnisverhütung  geburtenplanung  Geburtenkontrolle  Geburtenregelung  Schwangerschaftsverhütung  Antikonzeption  Kontrazeption | Verhütungsmittel  empfängnisverhütendes Mittel  Langzeitmethode  Kondom  Präservativ  Dreimonatsspritze  Intrauterinpessarm  Spirale | voorbehoedsmiddel  langwerkende anticonceptie  anticonceptiemiddelen  contraceptiva |
| **Abortion** | abortion  menstrual regulation | MR  termination of pregnancy | avortement | Aborto | Schwangerschaftsabbruch  Schwangerschaftsunterbrechung  Abtreibung | Abbruch  Kindesabtreibung | abortus  zwangerschap beëindigen  zwangerschap afbreken |
| **User desires** | desired family size  families of desired size  planned families  birth spacing  birth timing  reproductive therapy  st[eé]rilit[eéy] | unmet need  unintended pregnancy  unwanted pregnan  birth limiting  fertility regulation  unfp | r[eé]gulation de la f[eé]condit[eé]  r[eé]gulation de la fertilité[eé]  besoins non satisfaits  besoins non-satisfaits  nombre d'enfants souhait[eé]  nombre d'enfants id[eé]al  grossesse non pr[eé]vue  grossesse non planifi[eé]e  espacer  limiter  espacement des naissances | embarazo% no desead  embarazo% no planeado  espaciamiento de los nacimientos  espaciar los nacimientos  limitación de los nacimientos  limitar los nacimientos  tama[nñ]o deseado de% familia  regulación de la fecundidad  controlar la fecundidad | Geburtenabstand  Geburteneinschränkung  Geburtenregelung  Geburtenbeschränkungen  Fruchtbarkeitsregulierung | Gewünschte Kinderzahl  Gewünschte Familiengröße  Unerwünschte Schwangerschaft  ungeplante Schwangerschaft  ungewollte Schwangerschaft | gewenste gezinsgrootte  ongewenste zwangerschap  geboorte beperking  geboorte-beperking |
| **Family planning organisations** | Marie%Stopes  MSI  M. Stopes* | IPAS  BAPSA |  |  |  | |  |
| **Government policy and national goals** | reduce fertility  fertility reduc  reduced fertility  small%famil* | reduc%population growth*  decrease%family size*  demographic dividend | dividende d[eé]mographique | dividendo demográfico | demografische dividende | |  |
