# Supplementary material for: Donor funding for family planning: levels and trends between 2003 and 2013
Source: Health Policy Plan. 2018 Mar 9;33(4):574–82. doi: 10.1093/heapol/czy006 (PMC5894079; doi:10.1093/heapol/czy006)
Supplement: Supplementary Table 2 [file czy006_supplementary_table_2.docx]

Supplementary Table 2: Year-specific disbursements to family planning from all donors

| **Donor** | **2003** | **2004** | **2005** | **2006** | **2007** | **2008** | **2009** | **2010** | **2011** | **2012** | **2013** | **Total** |
| --- | --- | --- | --- | --- | --- | --- | --- | --- | --- | --- | --- | --- |
| **Bilateral** | **292.5** | **128.6** | **349.2** | **303.5** | **298.2** | **448.0** | **600.7** | **579.1** | **709.5** | **665.0** | **803.0** | **5177.1** |
| United States | 183.2 | 34.5 | 252.8 | 233.7 | 253.8 | 360.1 | 500.8 | 454.3 | 538.9 | 479.8 | 591.7 | 3883.6 |
| United Kingdom | 46.5 | 35.7 | 21.2 | 16.3 | 10.3 | 23.6 | 27.3 | 34.1 | 74.9 | 116.0 | 102.0 | 507.6 |
| Netherlands | 2.4 | 4.1 | 0.3 | 7.6 | 6.3 | 43.3 | 48.0 | 49.6 | 42.7 | 10.9 | 26.7 | 241.8 |
| Germany | 47.2 | 33.3 | 25.1 | 21.1 | 17.2 | 12.5 | 9.8 | 16.2 | 10.1 | 20.1 | 19.8 | 232.4 |
| Norway |  | 10.6 | 9.6 | 9.0 |  | 1.0 | 1.5 | 1.7 | 6.2 | 3.9 | 24.6 | 68.1 |
| Australia | 8.6 | 5.9 | 9.7 | 3.3 | 0.0 | 1.1 | 0.0 | 6.6 | 10.6 | 5.0 | 9.7 | 60.6 |
| Canada | 1.4 | 1.7 | 25.4 | 4.4 | 4.2 | 0.0 | 2.3 | 3.1 | 5.7 | 2.8 | 6.3 | 57.4 |
| Sweden | 1.2 | 1.1 | 3.8 | 5.9 | 4.3 | 2.4 | 6.0 | 3.2 | 3.6 | 20.4 | 4.8 | 56.8 |
| Denmark | 0.6 | 0.5 | 0.1 | 0.0 | 0.0 |  |  |  | 9.3 | 1.8 | 3.8 | 16.1 |
| Spain |  |  | 0.0 | 0.2 | 0.7 | 1.1 | 1.7 | 7.8 | 2.2 | 0.3 | 0.8 | 14.9 |
| Finland | 0.2 | 0.0 | 0.0 | 0.3 | 0.4 | 0.4 | 1.3 | 0.9 | 2.1 | 2.5 | 3.6 | 11.7 |
| France |  |  |  |  |  |  |  | 0.5 |  | 0.2 | 6.3 | 7.1 |
| Belgium |  | 0.9 | 1.1 | 0.9 | 0.8 | 0.0 | 0.4 | 0.0 | 0.4 |  | 0.5 | 5.0 |
| Korea |  |  |  |  | 0.1 | 1.6 | 1.2 | 0.6 | 0.1 | 0.6 | 0.2 | 4.4 |
| Luxembourg |  |  |  |  |  | 0.6 |  |  | 1.9 |  | 0.3 | 2.9 |
| Switzerland | 1.2 | 0.1 |  |  |  |  | 0.2 | 0.3 | 0.2 | 0.3 | 0.4 | 2.7 |
| New Zealand |  | 0.1 | 0.1 | 0.7 |  | 0.3 | 0.1 |  | 0.3 | 0.2 |  | 1.7 |
| Italy | 0.0 | 0.0 |  | 0.0 | 0.0 |  | 0.0 |  |  |  | 1.2 | 1.3 |
| Ireland |  | 0.1 | 0.0 | 0.1 | 0.0 |  | 0.0 | 0.2 |  |  | 0.1 | 0.5 |
| Iceland |  |  |  |  |  |  |  |  | 0.2 |  |  | 0.2 |
| Japan |  |  |  | 0.0 |  |  |  |  | 0.1 |  | 0.1 | 0.2 |
| Austria |  |  |  |  |  |  | 0.0 | 0.0 | 0.0 |  |  | 0.1 |
| Portugal |  |  |  |  |  |  |  |  |  |  | 0.1 | 0.1 |
| **Multilateral** | **109.6** | **8.9** | **11.1** | **0.9** | **0.9** | **3.8** | **11.1** | **0.6** | **2.3** | **44.3** | **36.7** | **230.0** |
| UNFPA | 96.8 | 1.1 | 0.6 | 0.9 | 0.8 | 2.1 | 0.9 |  |  | 25.7 | 29.6 | 158.4 |
| EU Institutions |  |  | 0.7 |  | 0.0 | 1.8 | 1.0 | 0.6 | 2.3 | 18.6 | 6.2 | 31.2 |
| UNICEF | 2.5 | 7.2 | 9.8 |  |  |  |  |  |  |  |  | 19.5 |
| IDA | 10.4 | 0.6 | 0.0 | 0.0 | 0.0 | 0.0 | 0.0 | 0.0 | 0.0 | 0.0 | 0.8 | 11.8 |
| AfDF |  |  |  |  |  |  | 9.1 |  |  |  |  | 9.1 |
| **Private donor** |  |  |  |  |  |  |  |  |  |  |  |  |
| Bill & Melinda Gates Foundation |  |  |  |  |  |  | 21.1 | 19.3 | 33.5 | 38.8 | 46.1 | 158.8 |
| **Total** | **402.2** | **137.4** | **360.2** | **304.4** | **299.0** | **451.8** | **632.8** | **599.0** | **745.3** | **748.1** | **885.7** | **5566.0** |
