# Supplementary material for: Donor funding for family planning: levels and trends between 2003 and 2013
Source: Health Policy Plan. 2018 Mar 9;33(4):574–82. doi: 10.1093/heapol/czy006 (PMC5894079; doi:10.1093/heapol/czy006)
Supplement: Supplementary Table 3 [file czy006_supplementary_table_3.docx]

Supplementary Table 3: ODA+ to family planning per woman and unmet need levels

|  | 2003 | | 2004 | | 2005 | | 2006 | | 2007 | | 2008 | | 2009 | | 2010 | | 2011 | | 2012 | | 2013 | |
| --- | --- | --- | --- | --- | --- | --- | --- | --- | --- | --- | --- | --- | --- | --- | --- | --- | --- | --- | --- | --- | --- | --- |
|  | $ | UNFP | $ | UNFP | $ | UNFP | $ | UNFP | $ | UNFP | $ | UNFP | $ | UNFP | $ | UNFP | $ | UNFP | $ | UNFP | $ | UNFP |
| Afghanistan | 0.03 | 29.6 | 0.00 | 29.6 | 0.00 | 29.7 | 0.00 | 29.6 | 0.00 | 29.4 | 5.39 | 29.3 | 13.61 | 29.1 | 6.35 | 29 | 6.37 | 28.6 | 6.94 | 28.4 | 1.93 | 28 |
| Albania | 1.05 | 9.7 | 0.23 | 10 | 0.00 | 10.3 | 0.51 | 11.5 | 1.10 | 12.7 | 3.95 | 14 | 3.54 | 13.8 | 2.67 | 13.6 | 1.08 | 13.4 | 4.25 | 13.2 | 0.00 | 13.1 |
| Algeria | 0.01 | 13.3 | 0.00 | 13 | 0.00 | 12.8 | 0.00 | 12.7 | 0.00 | 12.8 | 0.00 | 12.9 | 0.00 | 13 | 0.00 | 13.2 | 0.00 | 13.3 | 0.00 | 13.4 | 0.00 | 13.2 |
| Angola | 0.43 | 28.7 | 0.10 | 28.7 | 0.13 | 28.8 | 0.00 | 28.9 | 0.00 | 28.9 | 1.28 | 28.8 | 1.54 | 28.8 | 1.60 | 28.7 | 1.20 | 28.6 | 1.32 | 28.6 | 0.97 | 28.5 |
| Argentina | 0.01 | 13.9 | 0.00 | 12.8 | 0.00 | 13.2 | 0.00 | 13.7 | 0.00 | 14.2 | 0.00 | 14.7 | 0.00 | 15.2 | 0.00 | 15.7 | 0.00 | 16.2 | 0.00 | 16 | 0.00 | 15.8 |
| Armenia | 0.00 | 19.5 | 0.14 | 19.5 | 0.37 | 19.4 | 0.12 | 18.1 | 0.83 | 16.9 | 2.20 | 15.7 | 10.19 | 14.6 | 3.96 | 13.6 | 2.36 | 13.5 | 0.00 | 13.5 | 1.41 | 13.4 |
| Azerbaijan | 0.01 | 15.3 | 0.00 | 15.3 | 0.00 | 15.3 | 0.00 | 15.4 | 0.51 | 15.3 | 1.67 | 15.1 | 2.03 | 14.9 | 1.12 | 14.8 | 0.13 | 14.5 | 0.43 | 14.3 | 0.40 | 14.2 |
| Bangladesh | 2.32 | 15.9 | 0.01 | 15.4 | 2.09 | 15.6 | 1.07 | 15.9 | 0.35 | 16.2 | 0.49 | 15.5 | 0.49 | 14.8 | 0.58 | 14.2 | 0.48 | 13.6 | 0.59 | 13 | 0.73 | 12.7 |
| Belarus | 0.00 | 10.1 | 0.00 | 9.5 | 0.00 | 9 | 0.00 | 9.2 | 0.00 | 9.4 | 0.00 | 9.6 | 0.00 | 9.9 | 0.00 | 10.1 | 0.00 | 10.3 | 0.00 | 10.5 | 0.00 | 10.5 |
| Belize | 2.63 | 22.7 | 5.59 | 23.3 | 10.50 | 23.9 | 2.55 | 24.4 | 1.22 | 23 | 0.00 | 21.7 | 0.00 | 20.4 | 0.00 | 19.1 | 0.00 | 17.8 | 0.00 | 17.5 | 0.00 | 17.2 |
| Benin | 0.00 | 28.5 | 0.00 | 28.6 | 0.99 | 28.8 | 0.90 | 28.8 | 0.00 | 29.3 | 2.33 | 29.8 | 1.04 | 30.3 | 2.25 | 30.9 | 2.15 | 31.4 | 0.97 | 31.2 | 1.48 | 31 |
| Bhutan | 0.00 | 22.9 | 0.00 | 21.4 | 0.00 | 19.8 | 0.00 | 18.4 | 0.00 | 16.8 | 0.00 | 15.3 | 0.00 | 14 | 0.00 | 12.6 | 0.00 | 12.2 | 0.00 | 11.7 | 0.10 | 11.4 |
| Bolivia | 1.84 | 23.2 | 0.15 | 22.7 | 0.00 | 22.2 | 0.00 | 21.7 | 0.02 | 21.3 | 6.43 | 21 | 5.54 | 20.5 | 4.05 | 20 | 4.57 | 19.6 | 3.60 | 19.1 | 3.67 | 18.7 |
| Bosnia & Herzegovina | 0.00 | 17.3 | 0.00 | 17.7 | 0.16 | 18 | 0.16 | 18.3 | 0.10 | 18.1 | 0.06 | 17.8 | 0.01 | 17.6 | 0.00 | 17.4 | 0.00 | 17.3 | 0.00 | 17.1 | 0.11 | 17 |
| Botswana | 0.08 | 21.3 | 0.00 | 20.8 | 0.00 | 20.3 | 0.00 | 19.8 | 0.00 | 19.4 | 0.00 | 19 | 1.51 | 18.6 | 0.00 | 18.3 | 0.00 | 17.9 | 0.00 | 17.6 | 0.00 | 17.3 |
| Brazil | 0.00 | 8.4 | 0.00 | 8.1 | 0.02 | 7.8 | 0.01 | 7.4 | 0.01 | 7.4 | 0.00 | 7.5 | 0.00 | 7.5 | 0.00 | 7.5 | 0.00 | 7.5 | 0.00 | 7.6 | 0.00 | 7.6 |
| Burkina Faso | 0.59 | 29.3 | 0.03 | 28.7 | -0.03 | 28.1 | 0.00 | 27.5 | 0.00 | 27 | 0.00 | 26.4 | 0.00 | 25.8 | 0.13 | 25.2 | 0.03 | 25.6 | 0.75 | 25.9 | 1.02 | 26.3 |
| Burundi | 0.05 | 31.1 | 0.00 | 31.2 | 0.00 | 31.5 | 0.00 | 31.6 | 0.00 | 31.8 | 0.00 | 31.9 | 0.00 | 32 | 0.00 | 32.1 | 1.03 | 31.6 | 0.13 | 31.2 | 2.74 | 30.7 |
| Cambodia | 0.66 | 28.7 | 0.73 | 27.3 | 0.00 | 26 | 0.05 | 24 | 0.82 | 22.2 | 0.93 | 20.5 | 1.55 | 18.8 | 2.96 | 17.2 | 3.03 | 16 | 3.63 | 14.9 | 3.35 | 13.8 |
| Cameroon | 0.73 | 21.2 | 0.03 | 21 | 0.03 | 21.2 | 0.00 | 21.5 | 0.00 | 21.7 | 0.00 | 22 | 0.01 | 22.2 | 0.00 | 22.5 | 0.00 | 22.8 | 1.37 | 22.7 | 0.80 | 22.6 |
| Cabo Verde | 3.22 | 18 | 0.16 | 17.4 | -0.21 | 16.9 | 3.93 | 16.7 | 0.00 | 16.5 | 0.00 | 16.3 | 0.00 | 16.1 | 0.03 | 15.8 | 0.00 | 15.6 | 0.00 | 15.4 | 0.00 | 15.2 |
| Central African Republic | 0.41 | 22.3 | 0.03 | 22.5 | 0.58 | 22.7 | 0.00 | 22.8 | 0.00 | 22.9 | 0.00 | 23.1 | 0.00 | 23.2 | 0.00 | 23.4 | 0.00 | 23.4 | 0.27 | 23.3 | 0.15 | 23.3 |
| Chad | 0.71 | 20.4 | 0.33 | 20.5 | 0.10 | 20.9 | 0.01 | 21.2 | 0.01 | 21.5 | 1.04 | 21.8 | 0.00 | 22 | 0.00 | 22.3 | 0.00 | 22.5 | 0.00 | 22.7 | 0.02 | 22.8 |
| Chile | 0.00 | 15.4 | 0.02 | 15.2 | 0.00 | 14.9 | 0.00 | 14.7 | 0.00 | 14.5 | 0.00 | 14.4 | 0.00 | 14.2 | 0.00 | 14.1 | 0.00 | 13.9 | 0.00 | 13.7 | 0.00 | 13.6 |
| China | 0.01 | 2.9 | 0.00 | 3 | 0.00 | 3.1 | 0.00 | 3.2 | 0.00 | 3.3 | 0.01 | 3.4 | 0.00 | 3.4 | 0.00 | 3.5 | 0.00 | 3.5 | 0.00 | 3.6 | 0.00 | 3.7 |
| Colombia | 0.04 | 9.3 | 0.00 | 9 | 0.00 | 8.8 | 0.00 | 8.7 | 0.00 | 8.5 | 0.00 | 8.4 | 0.00 | 8.3 | 0.00 | 8.2 | 0.00 | 8.2 | 0.00 | 8.2 | 0.00 | 8.2 |
| Comoros | 2.05 | 32.9 | 0.00 | 32.6 | 0.00 | 32.5 | 0.00 | 32.2 | 0.00 | 32.1 | 0.68 | 31.9 | 0.00 | 31.8 | 0.00 | 31.7 | 0.00 | 31.5 | 0.22 | 31.4 | 0.34 | 31.2 |
| Congo | 0.00 | 21.4 | 0.00 | 21 | 0.00 | 20.5 | 0.00 | 20.1 | 0.02 | 19.7 | 0.00 | 19.4 | 0.00 | 19 | 0.00 | 18.6 | 0.00 | 18.2 | 0.00 | 18.1 | 0.02 | 18 |
| Cote d'Ivoire | 3.23 | 27 | 0.65 | 26.6 | 0.81 | 26.1 | 0.71 | 25.6 | 0.00 | 25.2 | 0.00 | 24.7 | 0.00 | 24.1 | 1.25 | 23.6 | 0.69 | 23 | 1.27 | 23.3 | 1.28 | 23.5 |
| Cuba | 0.11 | 9.9 | 0.35 | 9.7 | 0.19 | 9.5 | 0.13 | 9.3 | 0.09 | 9.1 | 0.00 | 8.9 | 0.00 | 8.8 | 0.00 | 8.6 | 0.01 | 8.6 | 0.00 | 8.6 | 0.02 | 8.6 |
| DPR Korea | 0.00 | 11.1 | 0.00 | 11.1 | 0.00 | 11 | 0.00 | 11 | 0.00 | 11 | 0.00 | 10.9 | 0.00 | 10.9 | 0.00 | 10.9 | 0.00 | 10.8 | 0.12 | 10.8 | 0.03 | 10.7 |
| DR Congo | 0.00 | 27.7 | 0.00 | 27.7 | 0.00 | 27.7 | 0.00 | 27.6 | 0.03 | 27.7 | 0.47 | 27.5 | 1.02 | 27.5 | 1.15 | 27.5 | 1.09 | 27.4 | 0.78 | 27.4 | 1.05 | 27.4 |
| Djibouti | 1.11 | 30.8 | 0.00 | 31 | 0.00 | 30.9 | 0.00 | 30.9 | 0.00 | 30.8 | 0.00 | 30.7 | 0.00 | 30.7 | 0.00 | 30.7 | 0.00 | 30.6 | 0.00 | 30.6 | 0.24 | 30.3 |
| Dominican Republic | 0.67 | 13.5 | 0.30 | 14 | 0.34 | 14.6 | 0.00 | 15.2 | 0.00 | 12.6 | 0.56 | 11.7 | 0.64 | 10.9 | 0.63 | 10.9 | 1.00 | 10.8 | 0.24 | 10.8 | 0.05 | 10.8 |
| Ecuador | 0.20 | 9.8 | 0.19 | 9.3 | 0.42 | 9.3 | 0.35 | 9.2 | 0.31 | 9.2 | 0.01 | 9.2 | 0.01 | 9.2 | 0.00 | 9.1 | 0.00 | 9.1 | 0.08 | 9.1 | 0.09 | 9.1 |
| Egypt | 0.73 | 12.2 | 1.83 | 12.2 | 1.76 | 12.2 | 1.85 | 12.1 | 1.90 | 11.9 | 2.30 | 11.8 | 1.00 | 11.9 | 0.65 | 12 | 0.17 | 12.1 | 0.05 | 12.2 | 0.03 | 12.3 |
| El Salvador | 0.34 | 15.1 | 0.00 | 14.6 | 0.00 | 14.2 | 0.00 | 13.8 | 0.00 | 13.3 | 1.63 | 13 | 1.41 | 12.8 | 1.20 | 12.6 | 2.05 | 12.4 | 2.06 | 12.2 | 0.36 | 12.1 |
| Equatorial Guinea | 0.41 | 32.2 | 0.00 | 32.3 | 0.00 | 32.3 | 0.00 | 32.5 | 0.00 | 32.6 | 0.00 | 32.8 | 0.00 | 33 | 0.00 | 33.2 | 0.00 | 33.4 | 0.00 | 33.2 | 0.00 | 33 |
| Eritrea | 0.08 | 28.8 | 0.00 | 28.9 | 0.00 | 29 | 0.00 | 29.1 | 0.00 | 29.2 | 0.00 | 29.3 | 0.00 | 29.3 | 0.00 | 29.2 | 0.00 | 29.2 | 0.00 | 29.2 | 0.02 | 29.1 |
| Ethiopia | 0.18 | 35.9 | 0.29 | 35.7 | 0.23 | 35.5 | 0.58 | 34 | 0.39 | 32.6 | 1.81 | 31.1 | 1.26 | 29.6 | 0.65 | 28 | 2.25 | 27.5 | 1.22 | 26.9 | 1.53 | 26.3 |
| Fiji | 1.43 | 20.6 | 0.00 | 20.5 | 1.35 | 20.4 | 0.00 | 20.3 | 0.00 | 20.1 | 0.06 | 20 | 0.13 | 19.9 | 0.00 | 19.7 | 0.00 | 19.6 | 0.00 | 19.5 | 0.00 | 19.4 |
| Macedonia | 0.25 | 18.2 | 0.05 | 18.3 | 0.17 | 18.4 | 0.00 | 18.5 | 0.00 | 18.5 | 0.00 | 18.6 | 0.00 | 18.7 | 0.00 | 18.9 | 0.00 | 19 | 0.10 | 18.8 | 0.43 | 18.6 |
| Gabon | 1.56 | 28.4 | 1.13 | 28.1 | 1.06 | 27.8 | 0.00 | 27.6 | 0.00 | 27.3 | 0.00 | 27.1 | 0.30 | 26.9 | 0.00 | 26.6 | 0.00 | 26.4 | 0.00 | 26.2 | 0.00 | 26 |
| Gambia | 1.35 | 28.9 | 0.38 | 28.8 | 0.18 | 28.7 | 0.47 | 28.6 | 0.00 | 28.5 | 0.00 | 28.4 | 0.00 | 28.3 | 0.00 | 28.1 | 0.00 | 28.2 | 0.00 | 28.1 | 0.05 | 28.1 |
| Georgia | 0.00 | 20.5 | 0.10 | 20.1 | 0.11 | 19.7 | 0.00 | 19.7 | 0.44 | 19.9 | 5.59 | 20.2 | 2.91 | 20.6 | 1.61 | 18.2 | 2.46 | 17.9 | 1.20 | 17.6 | 1.52 | 17.3 |
| Ghana | 0.13 | 35.7 | 0.00 | 35.9 | 0.06 | 36.1 | 0.00 | 36.4 | 0.01 | 36.4 | 1.00 | 36.4 | 2.19 | 35.6 | 1.90 | 34.9 | 3.14 | 34.2 | 1.79 | 34.3 | 4.55 | 34.4 |
| Guatemala | 0.86 | 24.3 | 0.00 | 23.4 | 0.00 | 22.5 | 0.01 | 21.7 | 0.15 | 20.8 | 2.22 | 20 | 4.36 | 19.6 | 2.63 | 19.1 | 1.78 | 18.8 | 1.83 | 18.4 | 2.83 | 18.1 |
| Guinea | 0.32 | 23.7 | 0.44 | 23.3 | 1.36 | 22.8 | 1.55 | 23.1 | 0.50 | 23.3 | 4.10 | 23.5 | 3.01 | 23.7 | 3.32 | 23.8 | 3.35 | 24 | 1.63 | 24.1 | 2.25 | 24.3 |
| Guinea-Bissau | 0.00 | 22.3 | 0.18 | 22.1 | 0.55 | 22 | 0.00 | 21.8 | 0.00 | 21.6 | 0.00 | 21.4 | 0.00 | 21.2 | 0.00 | 20.9 | 0.00 | 21.2 | 0.00 | 21.5 | 0.05 | 21.7 |
| Guyana | 2.13 | 30.1 | 2.68 | 30.3 | 4.25 | 30.6 | 0.00 | 30.9 | 0.00 | 30.6 | 0.00 | 30.3 | 0.00 | 30 | 0.00 | 29.3 | 0.00 | 28.8 | 0.00 | 28.2 | 0.00 | 27.6 |
| Haiti | 2.75 | 39.8 | 0.00 | 39.4 | 17.65 | 39.1 | 25.08 | 38.4 | 16.85 | 37.7 | 7.00 | 37.1 | 5.30 | 36.5 | 5.28 | 35.9 | 6.03 | 35.4 | 7.48 | 35 | 7.40 | 34.2 |
| Honduras | 0.05 | 16.9 | 0.41 | 16.7 | 0.32 | 16.5 | 0.00 | 15.4 | 0.02 | 14.4 | 2.40 | 13.5 | 2.11 | 12.6 | 2.40 | 11.8 | 1.44 | 11 | 1.85 | 10.9 | 1.91 | 10.8 |
| India | 0.12 | 15.9 | 0.00 | 15.3 | 0.01 | 14.8 | 0.02 | 14.9 | 0.07 | 15 | 0.06 | 14.7 | 0.07 | 14.4 | 0.10 | 14.1 | 0.15 | 13.9 | 0.13 | 13.7 | 0.17 | 13.5 |
| Indonesia | 0.44 | 14.4 | 0.19 | 14.3 | 0.26 | 14.2 | 0.02 | 14 | 0.00 | 13.8 | 0.00 | 13.3 | 0.03 | 12.8 | 0.00 | 12.4 | 0.00 | 12 | 0.03 | 11.6 | 0.04 | 11.5 |
| Iran | 0.02 | 8.5 | 0.03 | 8.2 | 0.00 | 7.9 | 0.00 | 7.6 | 0.00 | 7.3 | 0.00 | 7 | 0.00 | 6.8 | 0.00 | 6.5 | 0.00 | 6.5 | 0.01 | 6.5 | 0.02 | 6.5 |
| Iraq | 0.02 | 19 | 0.02 | 18.3 | 0.00 | 17.6 | 0.00 | 16.9 | 0.00 | 16.5 | 0.00 | 16.1 | 0.00 | 15.7 | 0.00 | 15.4 | 0.00 | 15 | 0.20 | 14.8 | 0.16 | 14.6 |
| Jamaica | 0.20 | 11.1 | 0.30 | 10.8 | 0.85 | 10.5 | 0.29 | 10.2 | 0.11 | 10 | 2.37 | 9.8 | 0.21 | 9.8 | 0.02 | 9.7 | 0.00 | 9.8 | 0.00 | 9.8 | 0.00 | 9.7 |
| Jordan | 0.25 | 15.3 | 0.04 | 15 | 0.74 | 14.7 | 0.04 | 14.4 | 0.00 | 14.1 | 14.98 | 13.9 | 13.76 | 13.8 | 12.92 | 13.2 | 14.72 | 12.6 | 15.64 | 12 | 18.68 | 12 |
| Kazakhstan | 0.04 | 15 | 0.00 | 15.6 | 0.00 | 16.2 | 0.00 | 16.8 | 0.00 | 16.7 | 0.05 | 16.7 | 0.00 | 16.7 | 0.00 | 16.7 | 0.03 | 16.4 | 0.08 | 16.2 | 0.06 | 16 |
| Kenya | 0.47 | 27.7 | 0.49 | 27.4 | 1.50 | 27.1 | 0.30 | 26.8 | 0.08 | 26.5 | 1.16 | 26.2 | 2.93 | 24.9 | 2.66 | 23.7 | 4.88 | 22.5 | 5.28 | 21.3 | 5.30 | 20.1 |
| Kyrgyz Republic | 0.35 | 14.6 | 0.00 | 14.9 | 0.06 | 15.3 | 0.00 | 15.7 | 0.00 | 16 | 0.91 | 16.4 | 0.71 | 16.7 | 0.00 | 17.1 | 0.29 | 17.4 | -0.05 | 17.6 | 0.19 | 17.4 |
| Lao PDR | 0.09 | 26.8 | 0.00 | 26.1 | 0.00 | 25.6 | 0.00 | 24.6 | 0.00 | 23.6 | 0.00 | 22.7 | 0.00 | 21.9 | 0.03 | 21 | 0.00 | 20.2 | 0.00 | 19.6 | 0.01 | 19 |
| Lebanon | 0.28 | 14.3 | 0.12 | 14.7 | 0.17 | 14.5 | 0.23 | 14.3 | 0.00 | 14.1 | 1.35 | 13.8 | 0.00 | 13.7 | 0.00 | 13.5 | 0.00 | 13.3 | 0.00 | 13.2 | 0.00 | 13 |
| Lesotho | 6.25 | 30.7 | 2.22 | 30.4 | 1.37 | 29 | 0.00 | 27.7 | 0.00 | 26.4 | 0.00 | 25.2 | 0.00 | 23.9 | 0.00 | 23 | 0.00 | 22 | 0.00 | 20.9 | 0.04 | 19.8 |
| Liberia | 0.47 | 34.3 | 0.00 | 34.4 | 0.00 | 34.6 | 0.00 | 34.9 | 0.00 | 35.1 | 7.16 | 34.5 | 11.89 | 34.1 | 11.25 | 33.6 | 11.64 | 33 | 6.34 | 32.5 | 8.85 | 31.9 |
| Madagascar | 2.34 | 25.1 | 0.32 | 24.3 | 0.05 | 23.4 | 0.00 | 22.5 | 0.04 | 21.6 | 1.50 | 20.5 | 1.10 | 20.3 | 2.76 | 20.1 | 1.17 | 19.9 | 3.44 | 19.6 | 2.97 | 19.4 |
| Malawi | 8.44 | 30.3 | 6.18 | 30 | 5.12 | 29.2 | 2.48 | 28.2 | 0.01 | 27.7 | 0.74 | 27.2 | 4.21 | 26.7 | 5.06 | 26.3 | 7.63 | 24.3 | 6.27 | 22.3 | 7.96 | 20.2 |
| Malaysia | 0.05 | 18 | 0.00 | 18.2 | 0.00 | 17.9 | 0.00 | 17.6 | 0.00 | 17.2 | 0.00 | 17 | 0.00 | 16.7 | 0.00 | 16.4 | 0.00 | 16.2 | 0.00 | 16 | 0.03 | 15.7 |
| Maldives | 2.97 | 26.1 | 0.00 | 26.3 | 0.00 | 26.7 | 0.00 | 27.1 | 0.00 | 27.6 | 0.00 | 28.1 | 0.00 | 28.6 | 0.00 | 28 | 0.00 | 27.3 | 3.52 | 26.7 | 3.78 | 26.1 |
| Mali | 0.27 | 29.4 | 0.01 | 29.1 | -0.01 | 28.7 | 0.00 | 28.4 | 0.05 | 28.1 | 2.41 | 27.8 | 2.46 | 27.4 | 3.29 | 27.1 | 3.71 | 26.8 | 2.54 | 26.4 | 5.51 | 26.6 |
| Mauritania | 0.00 | 31.6 | 0.00 | 31.6 | 0.00 | 31.5 | 0.00 | 31.5 | 0.00 | 31.4 | 0.00 | 31.3 | 0.00 | 31.3 | 0.00 | 31.2 | 0.00 | 31.2 | 0.58 | 31.2 | 0.29 | 31 |
| Mauritius | 0.00 | 6.5 | 0.00 | 6.5 | 0.00 | 6.5 | 0.00 | 6.5 | 0.00 | 6.5 | 0.00 | 6.5 | 0.00 | 6.5 | 0.00 | 6.5 | 0.00 | 6.5 | 0.00 | 6.5 | 0.00 | 6.6 |
| Mexico | 0.00 | 10.3 | 0.00 | 10.6 | 0.00 | 11 | 0.00 | 11.3 | 0.00 | 11.2 | 0.00 | 11 | 0.00 | 10.9 | 0.00 | 10.8 | 0.00 | 10.7 | 0.00 | 10.6 | 0.00 | 10.6 |
| Moldova | 0.19 | 11.7 | 0.11 | 11.9 | 0.25 | 12 | 0.00 | 12.1 | 0.00 | 12.3 | 0.00 | 12.4 | 0.00 | 12.5 | 0.00 | 12.6 | 0.00 | 12.8 | 0.33 | 13 | 0.18 | 12.9 |
| Mongolia | 0.66 | 9.1 | 0.00 | 9.7 | 0.00 | 10.4 | 0.00 | 11.6 | 0.00 | 13 | 0.00 | 14.4 | 0.00 | 14.7 | 0.00 | 15.1 | 0.00 | 15 | 0.75 | 14.8 | 0.62 | 14.8 |
| Montenegro | 0.00 | 18.6 | 0.00 | 19.5 | 0.00 | 20.4 | 0.00 | 20.8 | 0.00 | 21.3 | 0.00 | 21.8 | 0.01 | 22.3 | 0.00 | 22.7 | 0.00 | 23.2 | 0.00 | 23.6 | 0.00 | 24 |
| Morocco | 0.31 | 12.4 | 0.15 | 12 | 0.18 | 11.7 | 0.15 | 11.4 | 0.22 | 11.1 | 0.00 | 10.8 | 0.09 | 10.6 | 0.16 | 10.3 | 0.07 | 10.2 | 0.01 | 10 | 0.00 | 9.9 |
| Mozambique | 2.69 | 21.9 | 1.36 | 22.7 | 1.23 | 23.4 | 0.83 | 24.2 | 0.00 | 24.8 | 1.15 | 25.5 | 1.47 | 26.2 | 1.05 | 26.8 | 3.24 | 27.5 | 2.10 | 27.5 | 3.39 | 27.5 |
| Myanmar | 0.02 | 21.2 | 0.00 | 21 | 0.00 | 20.7 | 0.00 | 20.5 | 0.00 | 20.4 | 0.00 | 19.8 | 0.00 | 19.3 | 0.00 | 18.8 | 0.00 | 18.2 | 0.00 | 17.8 | 0.00 | 17.3 |
| Namibia | 0.00 | 22.6 | 0.00 | 22.2 | 0.02 | 21.8 | 0.00 | 21.5 | 0.00 | 20.7 | 1.23 | 20.1 | 1.46 | 19.5 | 0.00 | 19 | 0.00 | 18.5 | 0.00 | 18.1 | 0.00 | 17.6 |
| Nepal | 1.96 | 28.8 | 1.16 | 27.9 | 3.52 | 27.1 | 4.65 | 26.3 | 1.79 | 26.4 | 1.05 | 26.5 | 2.47 | 26.7 | 2.11 | 26.9 | 2.18 | 27.3 | 1.66 | 26.3 | 2.13 | 25.5 |
| Nicaragua | 8.53 | 13.1 | 0.12 | 12.3 | 4.17 | 11.6 | 2.45 | 11 | 0.91 | 10.1 | 3.16 | 9.3 | 2.16 | 8.6 | 2.65 | 7.9 | 5.12 | 7.3 | 2.85 | 7.2 | 2.03 | 7.2 |
| Niger | 0.05 | 17.6 | 0.10 | 17.4 | 0.04 | 17.2 | 0.45 | 16.9 | 0.00 | 16.9 | 1.46 | 16.9 | 0.51 | 16.8 | 0.16 | 16.8 | 0.13 | 16.7 | 0.03 | 16.5 | 0.71 | 16.9 |
| Nigeria | 0.32 | 18.3 | 0.05 | 18.8 | 0.28 | 19.3 | 0.13 | 19.8 | 0.40 | 20.2 | 0.52 | 20.7 | 0.86 | 20.9 | 0.50 | 21 | 0.85 | 21.2 | 0.83 | 21.4 | 1.41 | 21.6 |
| Oman | 0.00 | 32.2 | 0.00 | 32.6 | 0.03 | 33 | 0.00 | 33.5 | 0.00 | 34 | 0.00 | 33.3 | 0.00 | 32.5 | 0.00 | 31.7 | 0.00 | 31 | 0.00 | 30.3 | 0.00 | 29.6 |
| Pakistan | 0.63 | 26 | 0.07 | 26.2 | 0.06 | 26.3 | 0.10 | 25.8 | 2.37 | 25.3 | 0.02 | 24.4 | 0.78 | 23.5 | 0.77 | 22.7 | 0.66 | 21.8 | 0.81 | 20.9 | 1.47 | 20.8 |
| Panama | 0.06 | 18.4 | 0.00 | 18.5 | 0.00 | 18.8 | 0.00 | 18.9 | 0.00 | 19.2 | 0.00 | 19.5 | 0.00 | 19.8 | 0.00 | 18.7 | 0.00 | 17.7 | 0.00 | 16.8 | 0.00 | 15.9 |
| Papua New Guinea | 0.19 | 27.6 | 0.13 | 27.4 | 0.00 | 27.3 | 3.68 | 27.2 | 0.00 | 26.9 | 0.10 | 26.6 | 0.02 | 26.4 | 0.04 | 26.1 | 0.00 | 25.9 | 0.09 | 25.7 | 0.36 | 25.5 |
| Paraguay | 0.19 | 9.2 | 0.03 | 8.5 | 0.00 | 7.8 | 0.00 | 7.2 | 0.14 | 6.6 | 1.52 | 6.1 | 3.18 | 6.1 | 3.10 | 6.2 | 1.37 | 6.2 | 1.07 | 6.3 | 0.12 | 6.3 |
| Peru | 0.44 | 13.6 | 0.01 | 13.2 | 0.38 | 12.8 | 0.29 | 12.6 | 0.28 | 12.5 | 0.99 | 9.9 | 1.21 | 7.7 | 1.34 | 7 | 0.97 | 6.8 | 0.70 | 8.4 | 0.45 | 9.1 |
| Philippines | 0.98 | 22.6 | 0.23 | 22.3 | 2.93 | 22.2 | 2.22 | 22 | 1.03 | 22.2 | 1.31 | 22.4 | 1.19 | 21.7 | 1.10 | 21.1 | 1.61 | 20.5 | 1.05 | 19.4 | 1.19 | 18.3 |
| Rwanda | 0.66 | 36.7 | 0.02 | 36.7 | 0.04 | 36.9 | 0.07 | 34.4 | 0.18 | 31.1 | 5.07 | 28.4 | 3.45 | 25.4 | 2.04 | 22.3 | 2.52 | 21.8 | 4.11 | 21.3 | 4.96 | 20.9 |
| Samoa | 7.71 | 45 | 0.00 | 45.3 | 7.31 | 45.6 | 0.00 | 46 | 0.00 | 46.5 | 0.32 | 46.9 | 0.00 | 47.5 | 0.00 | 46.5 | 0.00 | 45.6 | 0.00 | 44.7 | 0.00 | 43.8 |
| Sao Tome & Principe | 0.00 | 38.4 | 0.00 | 38.7 | 0.00 | 38.9 | 0.00 | 39.1 | 0.00 | 38.8 | 0.00 | 38.5 | 0.00 | 37.5 | 0.00 | 36.6 | 0.00 | 35.7 | 0.00 | 34.9 | 2.03 | 34.2 |
| Senegal | 0.54 | 31.9 | 0.47 | 32 | 0.53 | 32.2 | 0.71 | 31.8 | 0.01 | 31.3 | 2.56 | 31 | 2.40 | 30.6 | 3.52 | 30.2 | 4.87 | 30.1 | 1.81 | 30 | 7.13 | 30 |
| Serbia | 0.12 | 13.6 | 0.12 | 14.3 | 0.06 | 14.9 | 0.64 | 14.4 | 0.11 | 13.9 | 0.00 | 13.4 | 0.00 | 12.9 | 0.00 | 12.4 | 0.00 | 12.7 | 0.00 | 12.9 | 0.02 | 13.1 |
| Sierra Leone | 0.74 | 28.1 | 0.00 | 28.2 | 0.00 | 28.3 | 0.00 | 28.4 | 0.00 | 28.6 | 0.00 | 28.8 | 0.57 | 28.3 | 0.37 | 27.8 | 1.59 | 27.2 | 0.02 | 26.6 | 0.11 | 26 |
| Solomon Islands | 2.88 | 22.7 | 0.00 | 22.5 | 2.61 | 22.4 | 0.00 | 22.2 | 0.00 | 22.2 | 0.28 | 22.1 | 0.00 | 22 | 0.00 | 21.9 | 3.25 | 21.8 | 1.58 | 21.7 | 1.13 | 21.6 |
| Somalia | 0.11 | 30.7 | 0.09 | 30.7 | 0.25 | 30.8 | 0.00 | 30.8 | 0.00 | 30.7 | 0.00 | 30.7 | 0.00 | 30.5 | 0.00 | 30.3 | 0.00 | 30.2 | 0.00 | 30 | 0.01 | 29.7 |
| South Africa | 0.07 | 14 | 0.00 | 13.8 | 0.00 | 13.7 | 0.00 | 13.5 | 0.00 | 13.3 | 0.00 | 13.2 | 0.14 | 13 | 0.08 | 12.9 | 0.05 | 12.7 | 0.32 | 12.6 | 0.02 | 12.5 |
| South Sudan | 0.00 | 29 | 0.00 | 29 | 0.00 | 29.1 | 0.00 | 29.1 | 0.00 | 29.1 | 0.00 | 29 | 0.00 | 29.1 | 0.00 | 29.1 | 5.24 | 29.3 | 3.86 | 29.4 | 2.61 | 29.5 |
| Sri Lanka | 0.15 | 8 | 0.00 | 7.7 | 0.00 | 7.4 | 0.00 | 7.2 | 0.00 | 7.2 | 0.00 | 7.2 | 0.00 | 7.3 | 0.00 | 7.3 | 0.00 | 7.3 | 0.00 | 7.4 | 0.00 | 7.4 |
| Sudan | 0.00 | 28.4 | 0.00 | 28.5 | 0.52 | 28.4 | 0.09 | 28.5 | 0.00 | 28.5 | 0.00 | 28.5 | 0.50 | 28.5 | 0.62 | 28.5 | 0.00 | 28.6 | 0.10 | 28.5 | 0.10 | 28.6 |
| Swaziland | 0.12 | 29.6 | 0.00 | 28.2 | 0.00 | 26.9 | 0.00 | 25.4 | 0.00 | 23.2 | 0.00 | 21 | 0.00 | 18.9 | 0.00 | 16.9 | 0.00 | 16.5 | 1.47 | 16.1 | 0.99 | 15.8 |
| Syria | 0.20 | 18.5 | 0.01 | 17.7 | 0.01 | 16.9 | 0.00 | 16.2 | 0.00 | 16.3 | 0.00 | 16.6 | 0.06 | 16.8 | 0.14 | 16.5 | 0.00 | 16.2 | 0.00 | 15.9 | 0.02 | 15.7 |
| Tajikistan | 0.00 | 21.6 | 0.00 | 21.4 | 0.06 | 21.2 | 0.00 | 21.2 | 0.00 | 21.4 | 0.58 | 21.6 | 0.70 | 21.8 | 0.70 | 22 | 0.25 | 22.2 | 0.53 | 22.5 | 0.37 | 22.3 |
| Tanzania | 0.15 | 24.4 | 0.03 | 25 | 0.18 | 25.2 | 0.00 | 25.4 | 0.27 | 25.6 | 0.97 | 25.7 | 4.23 | 25.9 | 3.83 | 25.4 | 2.95 | 24.9 | 4.21 | 24.4 | 3.65 | 23.9 |
| Thailand | 0.00 | 6.5 | 0.00 | 6.6 | 0.00 | 6.8 | 0.00 | 5.3 | 0.00 | 5.3 | 0.00 | 5.3 | 0.00 | 5.3 | 0.01 | 5.4 | 0.02 | 5.5 | 0.00 | 5.6 | 0.01 | 5.7 |
| Timor-Leste | 0.00 | 21.2 | 0.00 | 22.7 | 0.00 | 24.3 | 0.00 | 25.8 | 0.00 | 27.3 | 2.80 | 28.6 | 17.00 | 29.9 | 54.28 | 29.1 | 19.46 | 28.5 | 32.95 | 28 | 39.49 | 27.4 |
| Togo | 0.00 | 35.8 | 0.00 | 35.7 | 0.00 | 35.6 | 0.00 | 35.5 | 0.00 | 35.3 | 0.00 | 35.1 | 0.00 | 35 | 0.00 | 34.8 | 0.00 | 34.4 | 0.00 | 34.1 | 0.51 | 33.8 |
| Tonga | 13.02 | 29.1 | 0.00 | 29.1 | 12.24 | 29 | 0.00 | 29 | 2.16 | 29 | 0.56 | 29 | 0.00 | 28.9 | 0.00 | 28.9 | 0.00 | 28.9 | 0.00 | 28.9 | 0.00 | 28.6 |
| Tunisia | 0.17 | 11.4 | 0.00 | 11.4 | 0.00 | 11.5 | 0.00 | 11.6 | 0.00 | 11.4 | 0.00 | 11.2 | 0.02 | 11.1 | 0.00 | 11 | 0.00 | 10.8 | 0.00 | 10.7 | 0.00 | 10.7 |
| Turkey | 0.00 | 10 | 0.00 | 9.4 | 0.00 | 8.9 | 0.00 | 8.4 | 0.00 | 7.9 | 0.00 | 7.5 | 0.00 | 7.1 | 0.00 | 6.8 | 0.00 | 6.5 | 0.00 | 6.3 | 0.00 | 6 |
| Turkmenistan | 0.00 | 15.4 | 0.00 | 15.9 | 0.08 | 16.4 | 0.00 | 16.9 | 0.00 | 16.6 | 0.21 | 16.3 | 0.12 | 16.1 | 0.00 | 15.9 | 0.05 | 15.7 | 0.08 | 15.4 | 0.02 | 15.2 |
| Uganda | 0.08 | 37.5 | 0.04 | 37.9 | 0.08 | 38.2 | 0.00 | 38.6 | 0.00 | 37.8 | 1.06 | 37 | 1.33 | 36.3 | 3.49 | 35.7 | 8.04 | 35 | 11.38 | 34.7 | 9.22 | 34.4 |
| Ukraine | 0.00 | 10.1 | 0.00 | 10 | 0.00 | 9.8 | 0.00 | 10.1 | 0.00 | 10.4 | 0.17 | 10.3 | 0.41 | 10.3 | 0.37 | 10.3 | 0.33 | 10.2 | 0.27 | 10.1 | 0.27 | 10.2 |
| Uruguay | 0.28 | 7.4 | 0.11 | 7.5 | 0.00 | 7.5 | 0.00 | 7.5 | 0.00 | 7.5 | 0.00 | 7.5 | 0.00 | 7.5 | 0.00 | 7.5 | 0.00 | 7.5 | 0.34 | 7.5 | 0.33 | 7.5 |
| Uzbekistan | 0.13 | 9.7 | 0.13 | 9.7 | 0.37 | 9.8 | 0.13 | 9.9 | 0.02 | 9.9 | 0.00 | 9.8 | 0.04 | 9.7 | 0.00 | 9.7 | 0.01 | 9.7 | 0.03 | 9.6 | 0.02 | 9.6 |
| Vanuatu | 6.31 | 27 | 3.48 | 27 | 5.72 | 26.9 | 0.00 | 27 | 0.00 | 26.9 | 0.34 | 26.6 | 0.00 | 26.2 | 0.00 | 25.9 | 0.00 | 25.6 | 0.00 | 25.3 | 0.00 | 25 |
| Venezuela | 0.02 | 12.9 | 0.01 | 12.8 | 0.04 | 12.7 | 0.00 | 12.6 | 0.00 | 12.5 | 0.00 | 12.5 | 0.00 | 12.4 | 0.00 | 12.3 | 0.00 | 12.2 | 0.06 | 12.1 | 0.04 | 12.1 |
| Vietnam | 1.15 | 7.2 | 0.36 | 7 | 0.18 | 6.5 | 0.36 | 5.9 | 0.38 | 5.7 | 0.28 | 5.7 | 0.04 | 5.8 | 0.00 | 6 | 0.00 | 6 | 0.01 | 6.4 | 0.00 | 6.5 |
| West Bank & Gaza Strip | 0.68 | 18.3 | 0.02 | 18.3 | 0.01 | 18.2 | 0.01 | 18 | 0.00 | 17.6 | 0.00 | 17.3 | 0.00 | 16.9 | 0.00 | 16.6 | 0.00 | 16.4 | 0.00 | 16.1 | 0.47 | 15.9 |
| Yemen | 0.39 | 35.5 | 0.59 | 34.5 | 0.07 | 33.5 | 1.18 | 32.5 | 0.94 | 31.8 | 0.34 | 31.2 | 1.98 | 30.7 | 1.62 | 30.1 | 0.31 | 29.5 | 0.61 | 28.9 | 1.87 | 28.4 |
| Zambia | 2.54 | 27.7 | 0.02 | 27.7 | 0.10 | 27.6 | 0.01 | 27.5 | 0.00 | 27.5 | 2.07 | 26.4 | 2.74 | 25.3 | 3.77 | 24.2 | 4.93 | 23.2 | 3.65 | 22.2 | 9.81 | 21.1 |
| Zimbabwe | 2.95 | 16.2 | 3.15 | 16 | 1.93 | 15.9 | 1.11 | 15.6 | 0.03 | 15.2 | 0.65 | 14.9 | 4.27 | 14.6 | 3.48 | 14.4 | 6.32 | 13.6 | 4.21 | 12.8 | 0.98 | 12.1 |

UNFP = unmet need for family planning; $ = 2013 USD for family planning per woman aged 15–49
